# Government Guidance Fund Empowers the Supply Chain Financing Decision of Marine Ranching Considering Environmental Enrichment and Friendliness

**DOI:** 10.3390/ani13050897

**Published:** 2023-03-01

**Authors:** Xiaole Wan, Zhengwei Teng, Jilai Song, Yao Zhai, Kuncheng Zhang

**Affiliations:** 1 Management College, Ocean University of China, Qingdao 266100, China; 2 Marine Development Studies Institute of OUC, Key Research Institute of Humanities and Social Sciences at Universities, Ministry of Education, Qingdao 266100, China; 3 College of Continuing Education, Shandong University of Science and Technology, Qingdao 266590, China; 4 School of Marxism, Ocean University of China, Qingdao 266100, China

**Keywords:** government guidance fund, supply chain financing decision, environmental enrichment, marine ranching

## Abstract

**Simple Summary:**

The capital shortage for marine ranch construction will reduce their economic, ecological, and social benefits. By constructing the supply chain decision model to solve and analyze the results, we found that the change of the products’ environmental friendliness and the environmental enrichment degree of the marine ranching companies will affect the marine ranching construction level, and they are positively correlated. In addition, the enhancement of the guiding effect of government guidance fund can break through the dilemma of enterprise capital shortage and optimize the establishment and development level of marine ranching.

**Abstract:**

The construction of marine ranching is a concrete practice to fulfil the strategic objective of China’s maritime power. The shortage of funds has turned into an important issue to be resolved urgently in the modernization of marine ranching. This study constructs a supply chain system, involving a leading enterprise of marine ranching with short funds and a retailer, and introduces the government guidance fund to solve the issue of capital shortage. Then, we discuss the supply chain financing decision under two different power structure modes, and analyze the product environmental attribute (the product’s environmental friendliness and the environmental enrichment) and the guiding effect of government investment on the operation of different modes. The research shows that: (1) The wholesale price of products is mainly influenced by the dominant position of the marine ranching leading enterprise. Furthermore, the wholesale price and the marine ranching company’s profits increase with the growth of the product environmental attribute. (2) The retailer’s profit and the supply chain system’s profit are mainly affected by the dominant power of the retailer and are positively correlated with the product environmental attribute. In addition, the supply chain system’s overall profits are negatively related to the guiding effect of government investment.

## 1. Introduction

The waste of ocean resources and the disruption of the ocean environment make traditional fisheries face the dual constraints of resources and environment [1]. Marine ranching has attracted extensive attention from all countries since its appearance, and it has become a hot spot in the growth of the ocean economy as a new ecological fishery model [2]. Compared with traditional mariculture, marine ranching pays more attention to environment and quality, which not only reduces the pollution to the ecological environment, but also improves production efficiency. The “14th Five-Year Plan” of China proposed that we should adhere to land and sea coordination, promote marine ecological protection, marine economic development, marine rights protection, and accelerate the construction of a maritime power. According to Announcement No. 515 of the Ministry of Agriculture and Rural Affairs of the People’s Republic of China, as of mid-January, 2022, 153 national marine ranching demonstration zones have been approved, which indicates that the marine ranching industry has begun to take shape. China’s marine ranching has achieved a large-scale output in coastal provinces in recent years, but its construction and development are still in the primary stage. Due to marine ranching being a comprehensive system, its construction and development need to be considered comprehensively [3]. Marine ranching has a long construction period [4], and it belongs to a capital-intensive industry. Its establishment and development need more time and money, and the capital demand is large. China’s marine ranching leading enterprises are still in their infancy, and the financing difficulties caused by insufficient funds and broken a capital chain has become a bottleneck in the growth of marine ranching [4]. Thus, there is no time to delay in solving the dilemma of capital shortage in China’s marine ranching and building maritime power.

In view of the fund shortage for marine ranching establishment, the leading enterprises can adopt a variety of ways and channels to grow capitals. Enterprises can adopt supply chain finance (SCF) to ease the fund shortage. In the past few years, academic circles have paid more attention to SCF [5,6] and SCF has become a prospective solution to alleviate the financing problems of small and medium enterprises (SMEs) [7]. Marine ranching construction and operation involves many stakeholders, such as the government, enterprises, and fishermen, and needs overall planning and comprehensive management. The government, as a key subject, also plays a crucial role in alleviating the shortage of funds for leading enterprises in marine ranching. The government can not only support the operation and development of leading enterprises in marine ranching by formulating relevant policies [8], but also solve the financial constraints faced by enterprises through direct government subsidies and government guidance fund. Among them, the government guidance fund is an efficient way for the government to provide relief programs for the leading enterprises of marine ranching. Since marine ranching in China is a new industry, and the establishment scale of most leading enterprises in marine ranching is small, it belongs to start-ups [9]. The government’s shareholding in marine ranching through the government guidance fund can not only effectively ease the financing issues faced by the marine ranching leading companies as start-ups [10], but also effectively deal with the financing risks of enterprises in the process of SCF. In addition, the government guidance fund can also guide social capital through its guiding effect to jointly invest in marine ranching with a fund shortage. However, the existing research on solving the problem of fund shortages at the government level mostly focuses on the method of direct government subsidies, while the research on government guidance funding is relatively less.

There can be many diverse structures in the supply chain model. For the study of different power structures in a supply chain, scholars have conducted extensive discussions. For example, Luo et al. [11] constructed a retail supply chain model including manufacturers and retailers, and researched the impact of different power structures on the decision-making and profit of the supply chain. Li et al. [12] studied the impact of different power structures on products and their pricing decisions in the supply chain. Li and Mizuno [13] revealed the optimal pricing and inventory control strategy of a dual-channel supply chain under three different power structures. In addition, Zhai et al. [14] believed that the dominant power of manufacturers and retailers determines whether participants are leaders or followers in the market, which in turn affects the decision-making order. Therefore, how to make operational decisions based on different power structures to maximize their own benefits is an urgent problem for supply chain members. Motivated by this, this study focuses on the shortage of capital for marine ranching establishment. With “supply chain financing” and “government guidance fund” as the breakthrough points, a two-stage supply chain model including a leading enterprise of marine ranching and a retailer with a capital shortage is constructed. According to the dominant power of the marine ranching company and the retailer, the model can be divided into two modes, and the optimal solution under the two modes can be solved, respectively. In addition, the products’ environmental friendliness can protect the marine environment and environmental enrichment can effectively improve the behavior and benefits of marine organisms. Therefore, this study introduces the environmental enrichment variable into the model and this, combined with a product’s environmental friendliness variable, constitutes the product environmental attribute. Then, it analyzes the effect of the variable on the supply chain operation, and compares the decision outcomes for two different power structures. What is more, it provides a theoretical basis for the financing decision behaviors of the marine ranching supply chain, and offers relevant advice for solving the fund shortages of marine ranching.

According to the above analysis, the contribution of this paper can be divided into the following three points:

(i). The government guidance fund is applied to the marine ranching to study the influence of different power structures on supply chain operation. It broadens the research boundary of the government guidance fund.
(ii). Environmental enrichment and product’s environmental friendliness constitute the product environmental attribute of the marine ranching leading enterprise. The impact of this variable on the operation of the supply chain is analyzed.
(iii). Through parameter sensitivity analysis and comparative analysis of the two modes, relevant management suggestions are put forward from the perspective of the marine ranching leading company and the retailer, respectively.

## 2. Literature Review

This part expounds four kinds of literature flows based on the research themes and objectives: (1) marine ranching, (2) government guidance fund, (3) supply chain financing decision, and (4) environmental enrichment.

### 2.1. Marine Ranching

The notion of marine ranching comes from the practical experience of the scientific utilization of the ocean, and has steadily improved with the growth of fisheries. Marine ranching was first proposed by Japanese scholars in 1971, and it was defined as a system that can continuously produce food from marine biological resources. In 1979, Zeng Chengkui put forward “marine farming, marine pasture”, from which the notion of marine ranching in China originated [4]. The 230th Shuangqing Forum made clear the most authoritative definition so far, that is, marine ranching is an ecosystem that has the function of environmental protection and can realize the sustainable production of fishery in suitable sea areas. As a new fishery model [15], marine ranching has brought great economic and ecological benefits [2,16]. Marine ranching is a vital means for rich resources and ecological sustainable development [8]. It can not only make fishery production efficient, protect ocean environments, and realize the sustainable utilization of fishery resources [17], but also be a crucial fishery carbon sink producer [18]. Marine ranching’s development in China mainly relies on the guidance of the government and the participation of enterprises [19], which has become a major strategic issue of concern to the government. Recently, there have been many bottlenecks in the growth of marine ranching. The establishment of marine ranching was stagnant due to faulty laws and management systems [20], the urgent breakthrough of key technology [21], the low allocation efficiency of marine related resources [17], and fund shortages [22]. Modern marine ranching needs to consume more money in the construction process, so sufficient and sustainable funds are the necessary conditions to ensure the marine ranching establishment. Due to the long construction period of marine ranching [4], a large amount of funds need to be invested, and most of the marine ranching leading enterprises have a small construction scale, and their own funds and capital inflows are in a state of shortage. In the development process, enterprises will have the problem of capital chain rupture [4]. The construction level of marine ranching in China lags behind the strategic goal of accelerating the construction of a maritime power. Therefore, it is urgent to provide a financial relief plan for marine ranching.

### 2.2. Government Guidance Fund

The problem of fund shortages in marine ranching leading enterprises has many ways and channels to solve. SCF and other financing methods can raise more funds for enterprises [23]. The SCF is a novel financing model, which provides flexible financial products and services for upstream and downstream SMEs based on trust and the fair distribution of benefits around the core enterprises [24]. It can not only effectively solve problems such as liquidity pressure existing in traditional financing methods [25], but also optimize the financing performance of supply chain companies [26,27] and improve the benefits of all participants in the supply chain system [28]. However, SCF has some financing risks in the financing process, such as credit risk [29], trust risk [30], pledge risk [31], and moral hazard [32]. Furthermore, from the government level, government intervention is of great help to SMEs’ financing [33]; the problem of capital shortage can be solved through the government’s financial support for enterprises. Guo et al. [34] believes that with the participation of the government, enterprises will obtain more funds for financing activities. The government’s intervention supports the financing channels of enterprises and promotes opportunities for enterprises to obtain financing [35]. 

There are two ways for the government to ease the dilemma of company capital shortage: direct government subsidy and a government guidance fund [8]. On the one hand, marine ranching leading enterprises can use government subsidies to gain more economic benefits [36]. Government subsidies, as a coordination mode [37], provide financial support to enterprises in the supply chain, thus solving the financing difficulty caused by capital constraints [38]. It can also improve the output level and operation ability of enterprises [39], thereby improving the level of a supply chain system’s overall performance [40]. On the other hand, the government guidance fund is a valid way to guide the growth of start-ups. It is funded by the government and attracts investment from local governments, financial institutions, and social capital. It can not only provide financial support to start-ups, but also solve the financing risks (credit risk, pledge risk, etc.) generated in the process of supply chain financing [41]. The government guidance fund needs policy support and Cui et al. [42] constructed the evaluation indicator system of policy effectiveness from three dimensions, and believed that without the support of policy, the government could not guide the growth of funds. Compared with the method of direct government subsidies, the method of the government guidance fund tends to invest in start-ups. The government guidance funds’ aim is to inspire the growth of emerging industries and stimulate the vitality of social capital to make venture capital investment. The government guidance fund is a new way for the government to resolve the financing difficulties in the initial stage of companies. In addition, the government guidance fund gathers the advantages of the government and the market [10] and plays a guiding role, which can attract the investment of social capita and promote the upgrading and innovation of the industries [42]. The advantage of the government guidance fund is that it operates in a market-oriented way, which drives social capital to invest in areas encouraged and supported by the state. The literature review shows that the government guidance fund can effectively solve the initial financing difficulties faced by leading enterprises in marine ranching. However, the existing research on the government guidance fund to alleviate corporate financial constraints still needs to be strengthened.

### 2.3. Supply Chain Financing Decision

SCF is an effective way for companies to obtain operating capital by using supply chain cooperation relationships [43]. How decision makers make optimal decisions is an important issue, and supply chain financing can combine financial problems with operational decision-making problems [44]. Financing decision refers to the choice of the best solution for enterprises to raise funds and it is a key problem for the enterprises’ survival and growth. Currently, the supply chain system has become increasingly complex. Each subject in this system should make reasonable decisions to create value, reduce risk, and strengthen cooperation with each other in the system [45]. Many scholars have researched the financing decision of the supply chain from different situations. For example, Huang et al. [7] studied supply chain financing decisions under capital constraints; Shi et al. [46] analyzed the influence of reduced demand uncertainty on the financing decisions of supply chains constrained by capital; Zheng et al. [47] studied the impact of market uncertainty on supply chain financing decisions. In addition, Xia et al. [48] introduced cross-shareholding, and discussed the optimal decision behaviors of supply chain entities in diverse power structures. Also considering the effect of cross-shareholding on supply chain financing decisions are Fu and Ma [49], Zhang and Meng [50], etc. According to the literature review of supply chain financing decisions, we find that most scholars divided the supply chain system into two modes according to their different dominant positions, and discuss the optimal decision-making behaviors of different participants in diverse modes, for example Ren et al. [51], Xia et al. [48]. To sum up, the paper considers the effect of diverse power structures on marine ranching supply chain financing decisions under government guidance fund, and discusses the effect of product environmental attribute variable on the optimal decision-making result.

### 2.4. Environmental Enrichment

Environmental enrichment, which refers to a method of improving animal welfare and biological functions through a deliberate increase in environmental heterogeneity and complexity [52], has shown huge potential for meeting the requirements of both aquatic animals and human owners. It is an important requirement for improving farm animal welfare [53]. Environmental enrichment provides complexity that can benefit poultry welfare, including health and behavior, as well as emotional states [54]. Exposure to environmental enrichment affects various cognitive functions in animals [55]. That is, environmental enrichment can improve the cognitive development of animals and induce positive emotions [56]. In addition, environmental enrichment is also an experimental method, which refers to richer living conditions relative to the standard environment, including increasing sense, exercise, cognition, and social stimulation [57]. For the past few years, scholars have studied all kinds of animals and explored the influence of environmental enrichment on animals. For example, van der Staay et al. [58] explored the mechanism of environmental enrichment on pig decision-making behavior, and reached the conclusion that environmental enrichment can improve the learning ability and memory ability of animals. Alaniz et al. [59] demonstrated behavioral evolution based on environmental enrichment, and believed that environmental enrichment could significantly improve the welfare of toucans by stimulating food consumption and promoting exercise. Sumon et al. [60] explored the importance of environmental enrichment in animal reproduction, and believed that environmental enrichment will increase the reproduction rate of fish. The literature shows that the environmental enrichment can promote the improvement of animal behavior and increase animal benefits. In view of this, this study combines the environmental enrichment variable and the product’s environmental friendliness variable into the product environmental attribute variable, and discusses the effect of this variable on the optimal decision results.

In summary, based on the existing literature, this study introduced the government guidance fund to deal with the financing problem of marine ranching. Then, the effect of diverse power structures on supply chain operation were studied, which broadens the research boundary of the government guidance fund. In addition, the environmental enrichment variable is introduced into the study of the model to discuss the effect of this variable on the optimal decision results. Finally, the corresponding management enlightenment is put forward from the view of leading enterprises in marine ranching and retailers.

## 3. Methods

### 3.1. Problem Description

**Figure 1:**
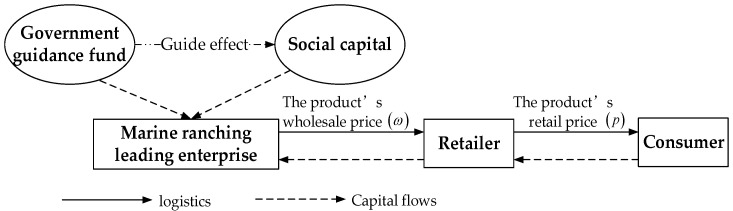
Basic model of marine ranching supply chain under government guidance fund.

The construction of marine ranching can not only develop and utilize marine biological resources reasonably and effectively, but also bring great social, economic, and ecological value. However, the marine ranching leading enterprises will face the shortage of funds in the progress of management and growth. As start-ups, the shortage of funds limits the operation and scale expansion of leading enterprises in marine ranching. The government, as a promoter of economic development, can take shares in marine ranching through government guidance funds, alleviate the constraints of the enterprise capital shortage, and then promote the continuous growth of marine ranching. Meanwhile, the government also promotes social capital into the marine ranching through its guiding effect, and then jointly solves the capital shortages of leading enterprises in marine ranching. Based on the description of the problem, this study constructs a supply chain system including a leading enterprise in marine ranching and a retailer. After the production process of the leading enterprise is completed, the enterprise sells the products related to marine living resources such as fish, shrimp, and shellfish to the retailer at a wholesale price ω, and then the retailer sells them to consumers at a retail price p. The basic model of marine ranching supply chain under government guidance fund is shown in Figure 1.

### 3.2. Basic Hypotheses

According to the difference of dominant power between the leading enterprise and the retailer in the supply chain system, two different modes are formed. In the two modes, there is a game relationship between the two sides. In the process of game, both sides constantly adjust their own strategies to seek optimal decisions. To facilitate analysis, the following hypothesis are put forward.

**Hypothesis** **1.** *The private capital of the leading enterprise in marine ranching is* $m_{0}$*, the government investment is* $I_{g}$*, and the initial investment of social capital is* $I_{0}$*. Considering the guiding role of government investment, the final investment of social capital is* $I_{s}=I_{0}\left(1+\sigma \right)$*, where* σ *is the guiding role of government investment. Considering that the government can drive social capital into the marine ranching leading enterprises through its guiding effect, the guiding role of government investment should meet* σ≥0.

**Hypothesis** **2.** *The product demand of the leading enterprise in marine ranching is* $q=a-bp+\alpha e_{s}$*, in which the market capacity is* a*,* p *is product price, and* a*,* p *are both >0.* b *is the product price coefficient, and* b>0*;* $e_{s}$ *is the product environmental attribute, which are influenced by the product’s environmental friendliness [16] and the marine ranching enterprise’s environmental enrichment [52], and meet* $e_{s}\geq 0$*;* α *is the influence coefficient of the product environmental attribute on demand,* α>0*; when the product* *’* *s environmental friendliness and the marine ranching enterprise’s environmental enrichment are improved, consumers will increase their purchase of the product. Considering the product demand should be greater than 0, so the product demand should meet* $a-bp+\alpha e_{s}>0$.

**Hypothesis** **3.** *The production cost per unit product of the leading enterprise in marine ranching is* c*, the financing cost is* $c_{I}$*, and* c>0*,* $c_{I}>0$.

**Hypothesis** **4.** *The proportion of government-owned marine ranching leading enterprise is* $\frac{I_{g}}{m_{0}+I_{g}+I_{s}}$*, the proportion of social capital-owned marine ranching leading enterprise is *$\frac{I_{s}}{m_{0}+I_{g}+I_{s}}$*, and the private capital ratio of the marine ranching leading enterprise is* $\frac{m_{0}}{m_{0}+I_{g}+I_{s}}$ *[61,62]. Therefore, it can be seen that the shareholding in the leading enterprise of marine ranching includes two parties. One is that the government shares in the leading enterprise through the government guidance fund. The other is that the social capital driven by the government guidance effect shares in the leading enterprise of marine ranching, and it is satisfied that* $0\leq \lambda_{1}\leq \bar{\lambda }$*,* $0\leq \lambda_{2}\leq \bar{\lambda }$*. Where* $\bar{\lambda }$ *represents the maximum proportion of equity that two entities can have. In order to ensure that both parties do not lose their control rights, it is necessary to meet the condition* $0\leq \bar{\lambda }\leq 0.5$ *[63], namely* $0\leq \lambda_{1}\leq 0.5$*,* $0\leq \lambda_{2}\leq 0.5$.

**Hypothesis** **5.** *The marine ranching company and the retailer are both risk-neutral. They all make their own deci-sions with the aim of maximizing their own benefits.*

Then, for the convenience of analysis, all the notations and the definition of the corresponding notations are listed in Table 1.

According to the above hypotheses, the profit of the leading enterprise and the retailer can be expressed, respectively:

(1) $$\pi_{m}=\frac{m_{0}}{m_{0}+I_{g}+I_{0}\left(1+\sigma \right)}\left(\omega -c\right)q-c_{I}$$

(2) $$\pi_{s}=\left(p-\omega \right)q$$

The profit of the supply chain system can be expressed as:

(3) $$\pi_{t}=\pi_{m}+\pi_{s}=\frac{m_{0}}{m_{0}+I_{g}+I_{0}\left(1+\sigma \right)}\left(\omega -c\right)q-c_{I}+\left(p-\omega \right)q$$

### 3.3. The Stackelberg Game Method

In the Stackelberg game model, the latter player can choose his own strategy according to the strategy of the first player, and the latter player’s strategy will also respond to the first player [64]. In the supply chain model constructed in the paper, different supply chain models are formed because the leading enterprise and the retailer occupy different leading positions in the supply chain system. Therefore, the optimal decision under each mode is different, and the strategies adopted will be different to some extent. The two different supply chain models proposed in this paper include the mode dominated by the marine ranching company and the mode dominated by the retailer. Taking the mode dominated by the marine ranching enterprise as an example, in this mode, the leading enterprise of marine ranching is the leader and the retailer is the follower, which satisfies the master–slave relationship in the Stackelberg game.

The backward induction is a method to solve the dynamic game equilibrium, which means that the actions of the participants in the game have a sequence, and the latter party can observe the actions of the former [65]. Because the supply chain decision-making model constructed in this paper follows the Stackelberg game model, and the backward induction is a commonly used method to solve the equilibrium solution in the Stackelberg game model. However, the backward induction is only suitable for finite step dynamic equilibrium, so it also has some limitations. Two different supply chain modes proposed in this paper include the mode dominated by the enterprise of marine ranching and the mode dominated by the retailer. The fourth part of this paper makes specific calculation and analysis on different supply chain decision-making modes. For ease of distinction, the two modes are marked as “Mode 1” and “Mode 2”, respectively.

## 4. Model Solving

### 4.1. Decision in Mode 1

**Figure 2:**
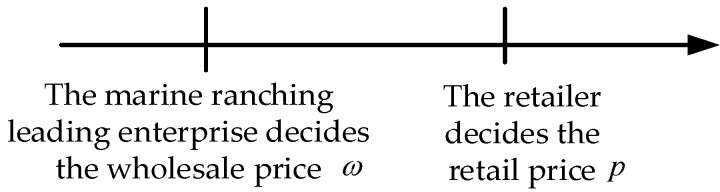
The game sequence of the leading enterprise and the retailer in Mode 1.

In this mode, the leading company of marine ranching occupies a dominant position. Then, the marine ranching leading enterprise first decides the product wholesale price ω, and then the retailer decides the product retail price p. The game sequence of the marine ranching leading enterprise and the retailer is shown in Figure 2.

Based on the backward induction method, the optimal decision-making problem of the marine ranching leading company and the retailer under this mode is solved. From Equation (1) and $q=a-bp+\alpha e_{s}$, the profit of the marine ranching leading company can be expressed as:

(4) $$\pi_{m}=\frac{m_{0}\left(\omega -c\right)\left(a-bp+\alpha e_{s}\right)}{m_{0}+I_{g}+I_{0}\left(1+\sigma \right)}-c_{I}$$

Similarly, the profit of the retailer can be expressed as:

(5) $$\pi_{s}=\left(p-\omega \right)\left(a-bp+\alpha e_{s}\right)$$

From Equation (5), the first and the second partial derivative of $\pi_{s}$ to p are calculated, respectively, to obtain $\frac{\partial \pi_{s}}{\partial p}=a-2bp+\alpha e_{s}+b\omega$, $\frac{\partial^{2}\pi_{s}}{\partial p^{2}}=-2b<0$. Therefore, $\pi_{s}$ is a strictly concave function about p, and based on $\frac{\partial \pi_{s}}{\partial p}=0$, the reaction function can be as follows:

(6) $$p=\frac{a+\alpha e_{s}+b\omega }{2b}$$

Substitute Equation (6) into $q=a-bp+\alpha e_{s}$, the product demand is:

(7) $$q=\frac{a+\alpha e_{s}-b\omega }{2b}$$

By substituting Equation (6) into Equation (4), the profit function of the leading enterprise in marine ranching is:

(8) $$\pi_{m}=\frac{m_{0}\left(\omega -c\right)\left(a-b\omega +\alpha e_{s}\right)}{2\left[m_{0}+I_{g}+I_{0}\left(1+\sigma \right)\right]}-c_{I}$$

From Equation (8), first order partial derivative and second order partial derivative of $\pi_{m}$ to ω are calculated, respectively, to obtain $\frac{\partial \pi_{m}}{\partial \omega }=\frac{m_{0}\left(a+\alpha e_{s}+bc\right)-2bm_{0}\omega }{2\left[m_{0}+I_{g}+I_{0}\left(1+\sigma \right)\right]}$, $\frac{\partial^{2}\pi_{m}}{\partial^{2}\omega }=\frac{-bm_{0}}{m_{0}+I_{g}+I_{0}\left(1+\sigma \right)}<0$. Therefore, $\pi_{m}$ is a strictly concave function about ω. Due to $\frac{\partial \pi_{m}}{\partial \omega }=0$, the optimal wholesale price of is:

(9) $$\omega =\frac{a+\alpha e_{s}+bc}{2b}$$

Substituting Equation (9) into Equation (7), the optimal of product’s retail price can be obtained:

(10) $$p=\frac{3a+3\alpha e_{s}+bc}{4b}$$

Substituting Equation (10) into $q=a-bp+\alpha e_{s}$, the product demand is:

(11) $$q=\frac{a+\alpha e_{s}-bc}{4b}$$

Equations (9) and (10) are substituted into Equations (4) and (5), respectively, to solve the optimal profits of the leading company and the retailer.

### 4.2. Decision in Mode 2

**Figure 3:**
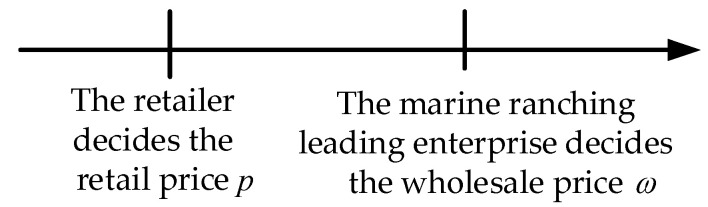
The game sequence of the leading enterprise and the retailer in Mode 2.

In this mode, the sales dominance is in the hands of the retailer, who occupies a dominant position in the supply chain system. The retailer’s position is higher than that of the marine ranching leading enterprise, and it occupyies a dominant position in the supply chain system. Meanwhile, the retailer first decides the product’s retail price p, and then the marine ranching leading enterprise decides the wholesale price ω. The game sequence of the leading enterprise and the retailer is shown in Figure 3.

Then, the optimal decisions of the leading enterprise and the retailer under this mode are solved by the backward induction method. For simplicity of calculation, we assume that the expected return of the retailer is θ, then:

(12) p=ω+θ

By substituting the Equation (12) into the Equations (4) and (5), the profit of the leading enterprise and the retailer can be expressed, respectively:

(13) $$\pi_{m}=\frac{m_{0}\left(\omega -c\right)\left(a-b\omega -b\theta +\alpha e_{s}\right)}{m_{0}+I_{g}+I_{0}\left(1+\sigma \right)}-c_{I}$$

(14) $$\pi_{s}=\theta \left(a-b\omega -b\theta +\alpha e_{s}\right)$$

According to Equation (13), the first order partial derivative and the second order partial derivative of $\pi_{m}$ to ω are calculated, respectively, to obtain $\frac{\partial \pi_{m}}{\partial \omega }=\frac{m_{0}\left(a-2b\omega -b\theta +\alpha e_{s}+bc\right)}{m_{0}+I_{g}+I_{0}\left(1+\sigma \right)}$, $\frac{\partial^{2}\pi_{m}}{\partial^{2}\omega }=\frac{-2bm_{0}}{m_{0}+I_{g}+I_{0}\left(1+\sigma \right)}<0$. Therefore, $\pi_{m}$ is a strictly concave function about ω. In view of $\frac{\partial \pi_{m}}{\partial \omega }=0$, we can have:

(15) $$\omega =\frac{a-b\theta +\alpha e_{s}+bc}{2b}$$

Substituting Equation (15) into Equation (14), we can obtain:

(16) $$\pi_{s}=\frac{a\theta -b\theta^{2}+\alpha e_{s}\theta -bc\theta }{2}$$

Based on Equation (16), the first order partial derivative and the second order partial derivative of $\pi_{s}$ to θ are calculated, respectively, obtained $\frac{\partial \pi_{s}}{\partial \theta }=\frac{a+\alpha e_{s}-bc}{2}-b\theta$, $\frac{\partial^{2}\pi_{s}}{\partial \theta^{2}}=-b<0$, so $\pi_{s}$ is a strictly concave function about θ. Due to $\frac{\partial \pi_{s}}{\partial \theta }=0$, we can obtain:

(17) $$\theta =\frac{a+\alpha e_{s}-bc}{2b}$$

Substituting Equation (17) into Equation (15), the optimal wholesale price of product is:

(18) $$\omega =\frac{a+\alpha e_{s}+3bc}{4b}$$

Substituting Equations (17) and (19) into Equation (12), the optimal retail price of product is:

(19) $$p=\frac{3a+3\alpha e_{s}+bc}{4b}$$

Substituting Equation (19) into $q=a-bp+\alpha e_{s}$, the product demand is:

(20) $$q=\frac{a+\alpha e_{s}-bc}{4}$$

Equations (17) and (18) are substituted into Equations (13) and (14), respectively, to solve the optimal profits of the leading company and the retailer.

## 5. Discussion

### 5.1. Conclusion of Equilibrium Results in Different Modes

According to Section 4.1 and Section 4.2, we can separately obtain the optimal solution in two different dominant modes, obtained Conclusions 1 and 2.

**Conclusion** **1.** *In the mode dominated by the marine ranching leading company:*

*The optimal product demand is* $q^{1*}=\frac{a+\alpha e_{s}-bc}{4b}$.

*The optimal retail price of the product is* $p^{1*}=\frac{3a+3\alpha e_{s}+bc}{4b}$.

*The optimal wholesale price of the product is* $\omega^{1*}=\frac{a+\alpha e_{s}+bc}{2b}$.

*The optimal profits of the retailer is* $\pi_{s}{}^{1*}=\frac{{\left(a+\alpha e_{s}-bc\right)}^{2}}{16b}$.

*The optimal profits of the leading enterprise in marine ranching is* $\pi_{m}{}^{1*}=\frac{m_{0}{\left(a+\alpha e_{s}-bc\right)}^{2}}{8b\left[m_{0}+I_{g}+I_{0}\left(1+\sigma \right)\right]}-c_{I}$.

*The overall optimal profits of the supply chain system is* $\pi_{t}{}^{1*}=\pi_{s}{}^{1*}+\pi_{m}{}^{1*}=\frac{{\left(a+\alpha e_{s}-bc\right)}^{2}}{16b}+\frac{m_{0}{\left(a+\alpha e_{s}-bc\right)}^{2}}{8b\left[m_{0}+I_{g}+\left(1+\sigma \right)\right]}-c_{I}$.

**Conclusion** **2.** *In the mode dominated by the retailer:*

*The optimal product demand is* $q^{2*}=\frac{a+\alpha e_{s}-bc}{4b}$.

*The optimal retail price of the product is* $p^{2*}=\frac{3a+3\alpha e_{s}+bc}{4b}$.

*The optimal wholesale price of the product is* $\omega^{2*}=\frac{a+\alpha e_{s}+3bc}{4b}$.

*The optimal profits of the retailer is* $\pi s^{2*}=\frac{{\left(a+\alpha e_{s}-bc\right)}^{2}}{8b}$.

*The optimal profits of the leading enterprise in marine ranching is* $\pi_{m}{}^{2*}=\frac{m_{0}{\left(a+\alpha e_{s}-bc\right)}^{2}}{16b\left[m_{0}+I_{g}+I_{0}\left(1+\sigma \right)\right]}-c_{I}$.

*The overall optimal profits of the supply chain system is* $\pi_{t}{}^{2*}=\pi_{s}{}^{2*}+\pi_{m}{}^{2*}=\frac{{\left(a+\alpha e_{s}-bc\right)}^{2}}{8b}+\frac{m_{0}{\left(a+\alpha e_{s}-bc\right)}^{2}}{16b\left[m_{0}+I_{g}+I_{0}\left(1+\sigma \right)\right]}-c_{I}$.

In order to facilitate the comparative analysis of diverse dominant modes, we list the equilibrium results for the two dominant modes in Table 2.

### 5.2. Comparison of Equilibrium Results in Different Modes

This part compares and analyzes the optimal solutions of the two modes (Conclusion 1 and Conclusion 2) in the supply chain system, and draws Conclusions 3–8.

**Conclusion** **3.** *In the two modes, the wholesale price of product has a relationship of* $\omega^{2*}<\omega^{1*}$*;* $\omega^{1*}$*,* $\omega^{2*}$ *increase with the growth of* $e_{s}$.

**Proof.**  From $q=a+\alpha e_{s}-bp>0$, we know $a+\alpha e_{s}>bp>bc$, and b>0. According to Equations (9) and (18), we have,

$\omega^{1*}-\omega^{2*}=\frac{a+\alpha e_{s}-bc}{4b}>0$.

In addition, the first partial derivatives of $\omega^{1*}$ and $\omega^{2*}$ to $e_{s}$ are calculated, respectively, and we can obtain,

$\frac{\partial \omega^{1*}}{\partial e_{s}}=\frac{\alpha }{2b}>0$; $\frac{\partial \omega^{2*}}{\partial e_{s}}=\frac{\alpha }{4b}>0$. □

As seen in Conclusion 3, the products’ wholesale price is mainly influenced by the dominant position of the marine ranching leading company in the supply chain system.

By comparing the mode dominated by the leading enterprise and the mode dominated by the retailer, we can see that when the leading enterprise dominates the supply chain system, to effectively guarantee its own profits maximization, the wholesale price set in the decision-making is higher. Under the retailer-led model, the retailer makes its own best decisions to make the profits optimal, which will decrease the product’s wholesale price as a retailer’s cost.

Under the two modes, with the growth of the product environmental attribute, the marine ranching leading company will increase wholesale price accordingly. This is because with the growth of $e_{s}$, the product’s environmental friendliness and the environmental enrichment increase and then the retailer’s demand for such products rises. Therefore, the leading enterprise of marine ranching will obtain profits by increasing wholesale prices.

**Conclusion** **4.** *In the two modes, the retail price of product has a relationship of* $p^{1*}=p^{2*}$*;* $p^{1*}$*,* $p^{2*}$ *increase with the growth of* $p^{2*}$.

**Proof.**  From Equations (10) and (19), we can obtain:

$p^{1*}-p^{2*}=0$.

In addition, the first partial derivatives of $p^{1*}$ and $p^{2*}$ to $e_{s}$ are calculated, respectively, and we can obtain:

$\frac{\partial p^{1*}}{\partial e_{s}}=\frac{\partial p^{2*}}{\partial e_{s}}=\frac{3\alpha }{4b}>0$. □

From Conclusion 4, it can be concluded that the retail price of products has nothing to do with the dominant position of all participants in different supply chain modes. 

Under the mode dominated by the marine ranching leading company and the mode dominated by the retailer, the retail price of products set by the retailer is the same. This indicates that regardless of the retailer’s position in the supply chain system, it will not affect the product retail price. 

In these two modes, the price formulated by the retailer is related to $e_{s}$. With the growth of $e_{s}$, the products’ retail price rises. For one thing, the growth in the product environmental attribute will rise the wholesale price (from Conclusion 3), and the retailer’s costs will rise. To reduce costs, the retailer passes on some costs to consumers by raising retail prices. For another thing, the growth of the product environmental attribute will increase consumer demand for the product. In a period of time, the retailer will raise retail prices due to the phenomenon of “oversupply”.

**Conclusion** **5.** *In the two modes, product demand has a relationship of* $q^{1*}=q^{2*}$*;* $q^{1*}$*,* $q^{2*}$ *increase with the growth of* $e_{s}$.

**Proof.**  From Equations (11) and (20), we can obtain:

$q^{1*}-q^{2*}=0$.

In addition, the first partial derivatives of $q^{1*}$ and $q^{2*}$ to $e_{s}$ are calculated, respectively, and we can obtain:

$\frac{\partial q^{1*}}{\partial e_{s}}=\frac{\partial q^{2*}}{\partial e_{s}}=\frac{\alpha }{4b}>0$. □

As with Conclusion 4, the product demand has nothing to do with the dominant position of all participants in diverse supply chain modes.

As shown in the two different modes, no matter which subject is dominant in the supply chain system, the product demand in optimal decision-making is always the same. This is because, in these two modes, the retail price of the product remains consistent (Conclusion 4 shows $q^{1*}=q^{2*}$), and consumers’ demand for the product will not change.

The change of product demand is connected with the change of $e_{s}$. With the growth of $e_{s}$, consumers’ demand for the product will increase. This is because, when $e_{s}$ growth, the product’s environmental friendliness and the environmental enrichment will increase. When other factors remain unchanged, consumers will increase the purchase and use of the product.

**Conclusion** **6.** *In the two modes, the profits of the retailer have a relationship of* $\pi_{s}{}^{1*}<\pi_{s}{}^{2*}$*;* $\pi_{s}{}^{1*}$*,* $\pi_{s}{}^{2*}$ *increase with the growth of* $e_{s}$.

**Proof.**  From Conclusion 1 and Conclusion 2,

$\pi_{s}{}^{2*}-\pi_{s}{}^{1*}=\frac{{\left(a+\alpha e_{s}-bc\right)}^{2}}{16b}>0$.

In addition, because $a+\alpha e_{s}-bc>0$, the first partial derivative of $\pi_{s}{}^{1*}$ to $e_{s}$ are calculated, and we can obtain:

$\frac{\partial \pi_{s}{}^{1*}}{\partial e_{s}}=\frac{\alpha \left(a+\alpha e_{s}-bc\right)}{8b}>0$;

Similarly, the first partial derivative of $\pi_{s}{}^{2*}$ to $e_{s}$ are calculated, and we can obtain:

$\frac{\partial \pi_{s}{}^{2*}}{\partial e_{s}}=\frac{\alpha \left(a+\alpha e_{s}-bc\right)}{4b}>0$. □

From Conclusion 6, it is concluded that the profits of the retailer mainly depend on its position in the supply chain system.

Comparing the two modes, it can be concluded that under the retailer leading supply chain mode, the retailer gains higher profits. This is because, although the retail price in the two modes is the same (Conclusion 4 shows $p^{1*}=p^{2*}$), the wholesale price of products in the retailer leading supply chain mode is lower (Conclusion 3 shows $\omega^{2*}<\omega^{1*}$). Therefore, for the retailer, they bear lower costs and gain higher profits.

In these two modes, the profits of a retailer’s optimal decision increases with the growth of $e_{s}$. This is because, with the growth of $e_{s}$, consumers’ product demand increases, the retail price of products formulated by retailers rises, and then the profits of the retailer increase.

**Conclusion** **7.** *In the two modes, the profits of the marine ranching leading enterprise have a relationship of* $\pi_{m}{}^{2*}<\pi_{m}{}^{1*}$*;* $\pi_{m}{}^{1*}$*,* $\pi_{m}{}^{2*}$ *increase with the growth of* $e_{s}$ *and decrease with the growth of* σ.

**Proof.**  From Conclusions 1 and 2,

$\pi_{m}{}^{1*}-\pi_{m}{}^{2*}=\frac{m_{0}{\left(a+\alpha e_{s}-bc\right)}^{2}}{16b\left[m_{0}+I_{g}+I_{0}\left(1+\sigma \right)\right]}>0$.

In addition, because $a+\alpha e_{s}-bc>0$, the first partial derivative of $\pi_{m}{}^{1*}$ to $e_{s}$ are calculated, and we can obtain:

$\frac{\partial \pi_{m}{}^{1*}}{\partial e_{s}}=\frac{m_{0}\alpha \left(a+\alpha e_{s}-bc\right)}{4b\left[m_{0}+I_{g}+I_{0}\left(1+\sigma \right)\right]}>0$;

Similarly, the first partial derivative of $\pi_{m}{}^{2*}$ to $e_{s}$ are calculated, and we can obtain:

$\frac{\partial \pi_{m}{}^{2*}}{\partial e_{s}}=\frac{m_{0}\alpha \left(a+\alpha e_{s}-bc\right)}{8b\left[m_{0}+I_{g}+I_{0}\left(1+\sigma \right)\right]}>0$;

because $I_{s}=I_{0}\left(1+\sigma \right)$, so $\pi_{m}{}^{1*}=\frac{m_{0}{\left(a+\alpha e_{s}-bc\right)}^{2}}{8b\left[m_{0}+I_{g}+I_{0}\left(1+\sigma \right)\right]}-c_{I}$, the first partial derivative of $\pi_{m}{}^{1*}$ to σ are calculated, and we can obtain:

$\frac{\partial \pi_{m}{}^{1*}}{\partial \sigma }=-\frac{m_{0}{\left(a+\alpha e_{s}-bc\right)}^{2}}{8bI_{0}{\left[m_{0}+I_{g}+I_{0}\left(1+\sigma \right)\right]}^{2}}<0$;

similarly, $\frac{\partial \pi_{m}{}^{2*}}{\partial \sigma }=-\frac{m_{0}{\left(a+\alpha e_{s}-bc\right)}^{2}}{16bI_{0}{\left[m_{0}+I_{g}+I_{0}\left(1+\sigma \right)\right]}^{2}}<0$. □

From Conclusion 7, we can determine that the profits of the marine ranching leading company mainly depending on its dominance in the supply chain system.

In the mode dominated by the marine ranching company, the optimal decision-making of the enterprise obtains higher profits. This is because, in this mode, the leading enterprise will take advantage of its dominance in decision-making, and the product wholesale price is higher (Conclusion 3 shows $\omega^{2*}<\omega^{1*}$). Moreover, with the growth of $e_{s}$, it will stimulate the rise of the retail price and the wholesale price, making the profits of the enterprise show an upward trend.

In these two modes, the profits of the leading enterprise are also related to the guiding role of government investment σ. With the growth of σ, the profits of the enterprise decrease. This is mainly due to the government increasing the guidance of social capital and promoting social capital into marine ranching. The increase in social capital investment funds will make the proportion of the marine ranching leading enterprise’s private capital $\frac{m_{0}}{m_{0}+I_{g}+I_{s}}$ decreased, and then the financial level of the enterprise decreases, and eventually leads to the profits of the marine ranching leading company showing a downward trend.

**Conclusion** **8.** *In the two modes, the profits of the supply chain system has a relationship of* $\pi_{t}{}^{1*}<\pi_{t}{}^{2*}$*;* $\pi_{t}{}^{1*}$*,* $\pi_{t}{}^{2*}$ *increase with the growth of* $e_{s}$ *and decrease with the growth of* σ.

**Proof.**  From Conclusions 1 and 2,

$\pi_{t}{}^{2*}-\pi_{t}{}^{1*}=\frac{\left(I_{g}+I_{s}\right){\left(a+\alpha e_{s}-bc\right)}^{2}}{16b\left[m_{0}+I_{g}+I_{0}\left(1+\sigma \right)\right]}>0$.

In addition, because $a+\alpha e_{s}-bc>0$, the first partial derivative of $\pi_{t}{}^{1*}$ to $e_{s}$ are calculated, to obtain:

$\frac{\partial \pi_{t}{}^{1*}}{\partial e_{s}}=\frac{\alpha \left(a+\alpha e_{s}-bc\right)}{8b}+\frac{m_{0}\alpha \left(a+\alpha e_{s}-bc\right)}{4b\left[m_{0}+I_{g}+I_{0}\left(1+\sigma \right)\right]}>0$;

Similarly, $\frac{\partial \pi_{t}{}^{2*}}{\partial e_{s}}=\frac{\alpha \left(a+\alpha e_{s}-bc\right)}{4b}+\frac{m_{0}\alpha \left(a+\alpha e_{s}-bc\right)}{8b\left[m_{0}+I_{g}+I_{0}\left(1+\sigma \right)\right]}>0$;

because $I_{s}=I_{0}\left(1+\sigma \right)$, the first partial derivative of $\pi_{t}{}^{1*}$ to σ are calculated, to obtain:

$\frac{\partial \pi_{t}{}^{1*}}{\partial \sigma }=-\frac{m_{0}{\left(a+\alpha e_{s}-bc\right)}^{2}}{8bI_{0}{\left[m_{0}+I_{g}+I_{0}\left(1+\sigma \right)\right]}^{2}}<0$;

Similarly, $\frac{\partial \pi_{t}{}^{2*}}{\partial \sigma }=-\frac{m_{0}{\left(a+\alpha e_{s}-bc\right)}^{2}}{16bI_{0}{\left[m_{0}+I_{g}+I_{0}\left(1+\sigma \right)\right]}^{2}}<0$. □

It can be seen from Conclusion 8 that the supply chain system’s profits are mainly connected with the dominant position of the retailer.

In the mode dominated by the retailer, the product’s wholesale price is low (Conclusion 3 shows $\omega^{2*}<\omega^{1*}$), which will lead to more profits for the retailer under the premise that the retail price and the product demand are consistent (Conclusions 4 and 5 shows $p^{1*}=p^{2*}$, $q^{1*}=q^{2*}$). As a start-up enterprise, the shortage of capital restricts the operation and scale expansion of the enterprise. Therefore, for the supply chain system, the retailer’s profits occupy a major part.

In these two modes, the supply chain system’s profits will rise with the growth of $e_{s}$. When $e_{s}$ growth, the product demand will rise, making profits increase. In addition, the overall profits level of the supply chain system will also reduce with the growth of σ. This is mainly because with the growth of σ, the proportion of the leading company’s private capital decreases, which makes the profits of the enterprise decrease, and thus leads to the overall profits of the supply chain system decrease.

## 6. Numerical Analysis

To prove the above conclusions more intuitively, the optimal decisions under different modes are further analyzed. Through the application program of MATLAB R2021b, we simulated the decision-making behavior of all participants in two modes. In addition, the numerical values of the variables and parameters assigned to the model in this paper are consistent with the hypotheses.

**Figure 4:**
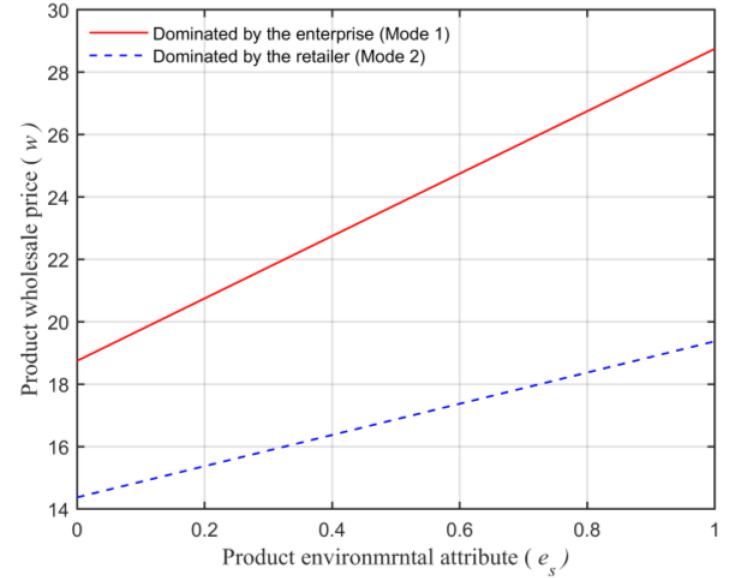
The relationship between the product environmental attribute and the product’s wholesale price.

**Figure 5:**
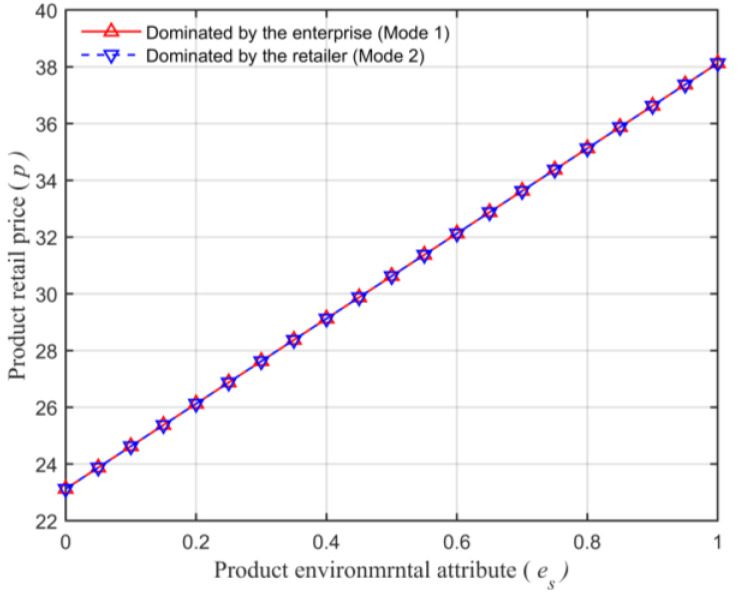
The relationship between the product environmental attribute and the product’s retail price.

**Figure 6:**
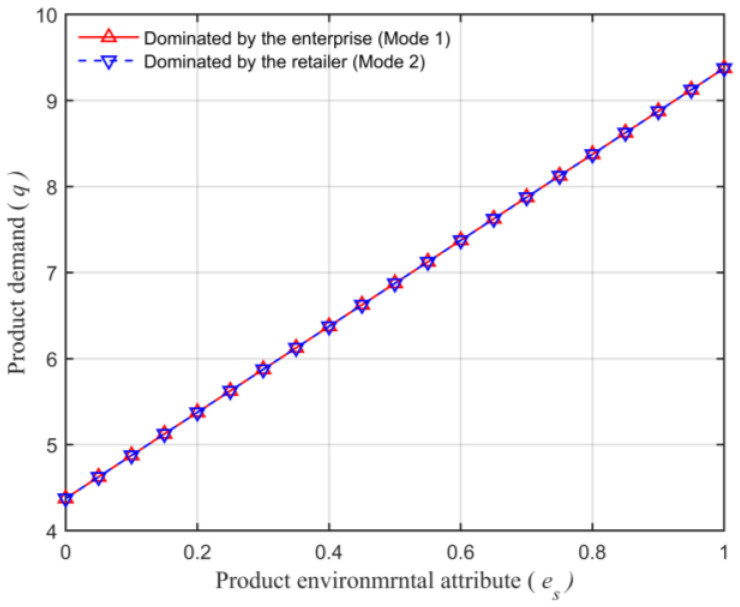
The relationship between the product environmental attribute and the product demand.

**Figure 7:**
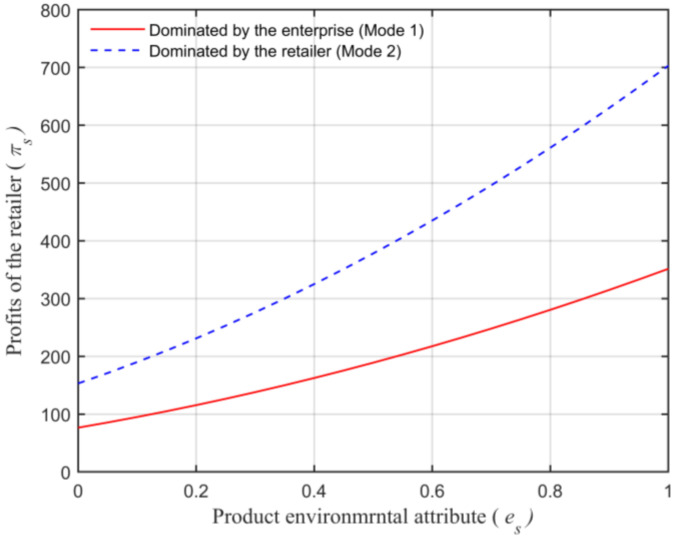
The relationship between the product environmental attribute and the retailer’s profits.

**Figure 8:**
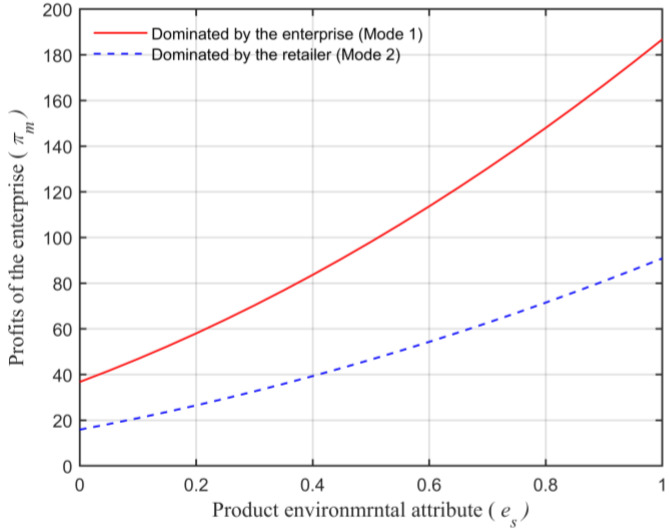
The relationship between the product environmental attribute and the marine ranching leading enterprise’s profits.

**Figure 9:**
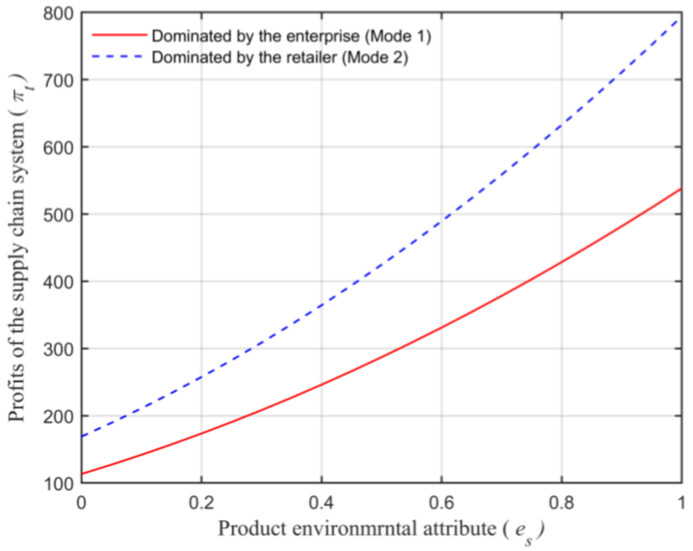
The relationship between the product environmental attribute and the supply chain system’s overall profits.

Assuming that the market capacity of the sales area a=110, the price coefficient of the product b=4, the impact coefficient of the product environmental attribute on demand α=80, the production cost of the unit product of the leading enterprise c=10, the financing cost of the unit product $c_{I}=5$, the private capital of the marine ranching leading enterprise $m_{0}=150$, the government investment $I_{g}=250$, and the initial investment of social capital $I_{0}=100$. Let the guiding role of government investment σ=0.5, and the product environmental attribute of the leading company $e_{s}$ is a variable, which changes in the interval [0,1] and makes the change images of each decision parameter and independent variable $e_{s}$. The comparison of a product’s wholesale price, a product’s retail price, product demand, the retailer’s profits, the marine ranching leading enterprise’s profit and the supply chain system’s profit under different modes are shown in Figure 4, Figure 5, Figure 6, Figure 7, Figure 8 and Figure 9.

From Figure 4, the product wholesale price is impacted by the dominant position of the leading company in the supply chain system. The product wholesale price made by enterprises is higher in the mode dominated by the marine ranching leading company, but lower in the mode dominated by the retailer. In addition, regardless of which mode, the wholesale price of products formulated by the leading enterprise will increase with the growth of $e_{s}$. This is consistent with Conclusion 3.

From Figure 5, the retail price is consistent in the two modes, indicating that the product retail price p is not significantly influenced by the dominant position of either subject. Moreover, from Figure 5, with the increase in the product environmental attribute $e_{s}$, the product retail price will also increase. This is consistent with Conclusion 4.

As can be seen in Figure 6, consumers’ demand for the marine ranching leading enterprise’s product is equal in either mode and consistent with the changing direction of $e_{s}$. This further illustrates that the product retail price and product demand size have a one-to-one correspondence. This is the same as in Conclusion 5.

Seen from Figure 7, under different modes, the size of the retailer’s profits meets the relationship of $\pi_{s}{}^{1*}<\pi_{s}{}^{2*}$, which further shows that the $\pi_{s}$ size of the retailer’s profits is connected with the size of the retailer’s dominant position in the supply chain system. Moreover, from Figure 7 that under any mode, the profits obtained by the retailer will increase with the growth of $e_{s}$. This is because, with the growth of $e_{s}$, consumers’ demand for high environmental friendliness and high environmental enrichment products will rise, the retail price of the product will also rise, which eventually leads to the increase in profits obtained by the retailer $\pi_{s}$. This is consistent with Conclusion 6.

As can be seen in Figure 8, the size of the profits of the leading company under the two modes satisfies $\pi_{m}{}^{2*}<\pi_{m}{}^{1*}$, which further illustrates that the profits’ size of the enterprise has a one-to-one correspondence with the size of the dominant power of the enterprise. Moreover, it can be seen in Figure 8 that the profits of the enterprise $\pi_{m}$ and the product environmental attribute $e_{s}$ change in the same direction. Therefore, it is consistent with Conclusion 7.

It can be seen from Figure 9 that, under the two modes, the relationship between the supply chain system’s overall profits satisfies $\pi_{t}{}^{1*}<\pi_{t}{}^{2*}$, which indicates that the profits are related to the dominant position of the retailer in the supply chain system. This is mainly due to the shortage of funds faced by the leading company in the initial stage of establishment, which limits their own development of the enterprise itself. In addition, as shown in Figure 9, the supply chain system’s profits rise with the growth of $e_{s}$. This is consistent with Conclusion 8.

**Figure 10:**
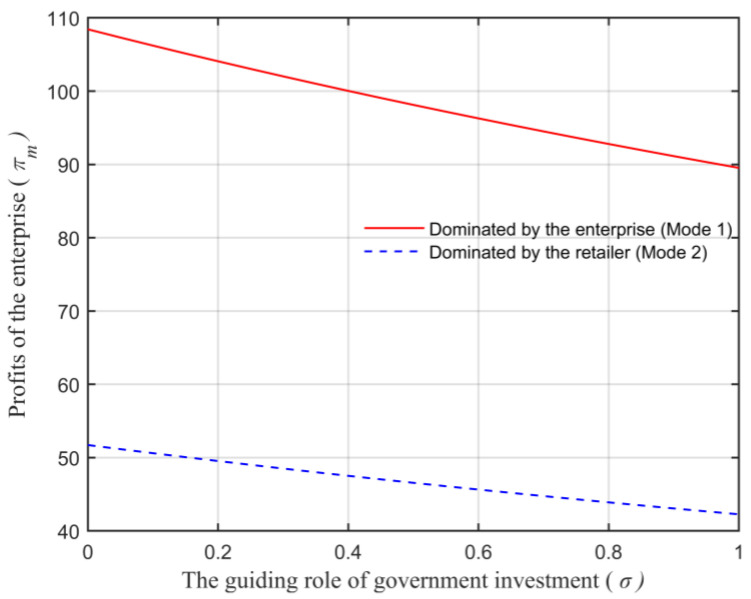
The relationship between the guiding role of government investment and the marine ranching leading enterprise’s profits.

**Figure 11:**
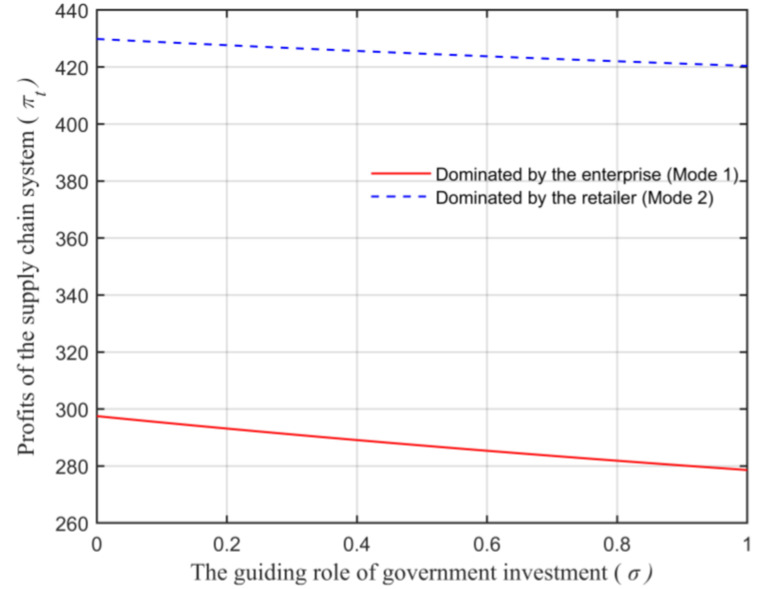
The relationship between the guiding role of government investment and the supply chain system’s overall profits.

Let the enterprise’s product environmental attribute $e_{s}=0.5$, and the guiding role of government investment as an independent variable change in the interval [0,1]. Under different modes, the changes of the marine ranching leading enterprise’s profits and the supply chain system’s overall profits with the guiding role of government investment σ are shown in Figure 10 and Figure 11.

From Figure 10, the profits of the enterprise are also related to the guiding role of government investment σ, and under the two modes, the profits of the marine ranching leading enterprise are inversely correlated with σ. This is because, with the growth in the guiding role of government investment σ, the ratio of the leading enterprise’s private funds will decrease, and ultimately lead to a downward trend in profits. This is consistent with Conclusion 7.

Figure 11 shows that the supply chain system’s overall profits level is also in connection with σ. With the growth of the government investment’s guiding role σ, the overall profits show a downward trend. This is because, σ is inversely proportional to the profits of the marine ranching leading company, which consistent with Conclusion 8.

To sum up, the results of numerical analysis in this section are consistent with the conclusions drawn by the model, which further supports the conclusions drawn in the study.

## 7. Conclusions and Implications

### 7.1. Conclusions

This study constructs a two-level supply chain system, including a leading marine ranching enterprise short of funds and a retailer, and the government guidance fund is introduced. Considering the difference in the dominant positions of the marine ranching leading enterprise and the retailer in the supply chain system, the mode dominated by the leading enterprise and the mode dominated by the retailer are constructed. Using the backward induction method in the game model, the optimal decisions of each subject under the two modes are calculated, and the optimal values in two different game modes are compared. Furthermore, we analyze the optimization results of the two modes in the application of MATLAB R2021b by numerical analysis. The research shows that:

(1) As far as the marine ranching leading companies are concerned, the wholesale price is mainly impacted by the dominance of the marine ranching leading company in the supply chain system. Generally speaking, there is a positive correlation between wholesale price and its dominant position. The products’ wholesale price is higher in the mode dominated by the marine ranching enterprise and lower in the mode dominated by the retailer. In addition, the optimal profits of the leading company are also related to its dominant position, and they are positively correlated. The marine ranching leading enterprises’ profits show the same trend with the environmental attributes of their products and decreases with the growth in the guiding role of government investment.

(2) For retailers, the products’ retail price is not significantly affected by the retailer’s dominant power. The product demand and the retail price are one-to-one correspondence in these two modes. The retail price is consistent in the two modes, and the demand also has an equal relationship in the two modes. The retailer’s optimal profits are positively related to its dominance in the supply chain system, which is higher in the mode dominated by the retailer and lower in the mode dominated by the marine ranching leading company. The product retail price, demand, and retailer’s profits will increase with the growth of the product environmental attribute. In addition, the overall profits of the supply chain system will also be affected by the retailer’s dominance. They are positively related to the environmental attribute of products and negatively related to the guiding role of government investment.

### 7.2. Management Implications

According to the conclusions of the last section, this section puts forward corresponding management enlightenment from the perspective of the marine leading companies and retailers. First, on the one hand, the leading companies of marine ranching can effectively integrate the concept of environmental friendliness into the whole process of the product production line by using green environmental protection materials [9] and then improving the production technology of products and developing the production process with more environmental protection concepts, so as to improve the products’ environmental friendliness produced by the leading enterprises. It can also make reasonable and effective use of green technologies, such as environmental enrichment, to improve the environmental enrichment degree of enterprises and stimulate consumers to buy so as to obtain a higher profit level [66]. In the meantime, if the leading enterprises of marine ranching can use a government guidance fund and social capital rational to enhance the environmental friendliness of products and the environmental enrichment, the willingness of governments to guide the fund will be improved. On the other hand, enterprises can also expand the sales of their products through a variety of marketing channels and increase the proportion of their own funds, so as to improve their financial level and operational capacity.

Second, retailers, as the downstream enterprises of the supply chain system, can stimulate consumers’ demand for highly environmental friendliness marine-related products by designing efficient marketing strategies to encourage consumers to purchase. In addition, retailers can also carry out product brand publicity activities through a variety of channels to convey information on the environmental friendliness of marine products and their environmental enrichment to potential consumers, and to enhance the brand image of products, so that consumers have a sense of identity and belonging to such products, and then the retailer’s own profits reach a high level.

Third, government, as a policy maker, can formulate relevant policies to enhance consumers’ awareness of purchasing environmental products. Moreover, the government can also establish an effective supervision mechanism and use public opinion to enhance consumers’ demand for environmental friendliness products produced by marine ranching leading enterprises, thereby enhancing the overall profit level of the leading enterprises, retailers, and supply chain systems. In addition, the government should effectively use the amplification effect of financial leverage and guide social capital to jointly invest in the start-up marine ranching leading companies so as to promote the operation of enterprises.

### 7.3. Future Research

This study discusses decision-making under different dominant modes, the action mechanism of product environmental attribute on the decision-making of the marine ranching leading company, and the guiding role of government investment in decision-making. The application of this model will help study the establishment and development of marine ranching from a multi-disciplinary perspective. 

However, this research can be further developed. Firstly, in this model, we only consider the influence of the product environmental attribute of the leading enterprise in marine ranching on the decision-making of the supply chain system. In the progress of actual management operations, in addition to the impact of the product environmental attribute on decision-making, other elements (such as advertising and public opinion) will also affect the decision-making system [67]. Secondly, the supply chain financing decision of marine ranching will also be influenced by potential factors such as citizen science initiatives and social media platforms. Citizen science can be clearly designed and incorporated into fishery decision-making management [68], which is helpful for the long-term sustainability of marine resources fishing. Similarly, as a supplementary form of the government, the social media can restrain and supervise the behavior of the marine ranching leading enterprises and enhance the fairness of marine ranching transactions [9]. Finally, this study introduces the government guidance fund, and through the guiding role of government investment, promotes social capital to jointly solve the dilemma of funds shortage of leading enterprises in marine ranching. However, according to Zheng et al. [69], although some coastal governments have established a government guidance fund, most of these marine industry funds are scattered, and investment and industrial scope are clearly limited and the scale of financing cannot meet the financial needs of marine ranching enterprises. These problems are the future research directions that need to be further expanded and improved. Future research should be further expanded and explored from the above two aspects, and the model can be enriched by introducing more influencing variables, such as advertising investment effort level [67], and the boundary of the constructed model can be expanded by increasing or reducing hypotheses. In addition, we can also consider the role of other parties (such as banks and insurance) in solving the shortage of funds in marine ranching construction.

## Figures and Tables

**Table 1:** List of notations.

Notations	Definition
Model Parameters	
$m_{0}$	The private capital of the leading company in marine ranching
$I_{g}$	The government investment
$I_{0}$	The initial investment of social capital
$I_{s}$	The final investment of social capital, $I_{s}=I_{0}\left(1+\sigma \right)$
σ	The guiding role of government investment, σ≥0
a	The market capacity, a>0
b	The product price coefficient, b>0
$e_{s}$	The product environmental attribute, $e_{s}>0$
α	The influence coefficient of the product environmental attribute on demand, α>0
c	The production cost per unit product
$c_{I}$	The financing cost
Decision Variables	
$p^{1}$, $p^{2}$	The product retail price
$\omega^{1}$,$\omega^{2}$	The product wholesale price
Functions	
q	The product demand
$\pi_{m}$	The profits of the enterprise
$\pi_{s}$	The profits of the retailer
$\pi_{t}$	The profits of the supply chain system

**Table 2:** Equilibrium results.

Mode	Dominated Be the Enterprise (Mode 1)	Dominated by the Retailer (Mode 2)
Product demand	$q^{1*}=\frac{a+\alpha e_{s}-bc}{4b}$	$q^{2*}=\frac{a+\alpha e_{s}-bc}{4b}$
Product’s retail price	$p^{1*}=\frac{3a+3\alpha e_{s}+bc}{4b}$	$p^{2*}=\frac{3a+3\alpha e_{s}+bc}{4b}$
Product’s wholesale price	$\omega^{1*}=\frac{a+\alpha e_{s}+bc}{2b}$	$\omega^{2*}=\frac{a+\alpha e_{s}+3bc}{4b}$
Profits of the retailer	$\pi_{s}{}^{1*}=\frac{{\left(a+\alpha e_{s}-bc\right)}^{2}}{16b}$	$\pi s^{2*}=\frac{{\left(a+\alpha e_{s}-bc\right)}^{2}}{8b}$
Profits of the enterprise	$\pi_{m}{}^{1*}=\frac{m_{0}{\left(a+\alpha e_{s}-bc\right)}^{2}}{8b\left[m_{0}+I_{g}+I_{0}\left(1+\sigma \right)\right]}-c_{I}$	$\pi_{m}{}^{2*}=\frac{m_{0}{\left(a+\alpha e_{s}-bc\right)}^{2}}{16b\left[m_{0}+I_{g}+I_{0}\left(1+\sigma \right)\right]}-c_{I}$
Profits of the supply chain system	$\begin{array}{l} \pi_{t}{}^{1*}=\frac{{\left(a+\alpha e_{s}-bc\right)}^{2}}{16b}-c_{I}\\ +\frac{m_{0}{\left(a+\alpha e_{s}-bc\right)}^{2}}{8b\left[m_{0}+I_{g}+\left(1+\sigma \right)\right]} \end{array}$	$\begin{array}{l} \pi_{t}{}^{2*}=\frac{{\left(a+\alpha e_{s}-bc\right)}^{2}}{8b}-c_{I}\\ +\frac{m_{0}{\left(a+\alpha e_{s}-bc\right)}^{2}}{16b\left[m_{0}+I_{g}+I_{0}\left(1+\sigma \right)\right]} \end{array}$

## Data Availability

Not applicable.
